# Genome-wide analysis of TCP transcription factor family in sunflower and identification of *HaTCP1* involved in the regulation of shoot branching

**DOI:** 10.1186/s12870-023-04211-0

**Published:** 2023-04-27

**Authors:** Yu Wu, Jianbin Zhang, Chaoqun Li, Xinyi Deng, Tian Wang, Lili Dong

**Affiliations:** College of Horticulture, Anhui Agricultural University, Changjiang Road, Hefei, 230036 Anhui China

**Keywords:** Sunflower, Shoot branching, TCP, Expression analysis, Functional analysis

## Abstract

**Background:**

Sunflower is an important ornamental plant, which can be used for fresh cut flowers and potted plants. Plant architecture regulation is an important agronomic operation in its cultivation and production. As an important aspect of plant architecture formation, shoot branching has become an important research direction of sunflower.

**Results:**

TEOSINTE-BRANCHED1/CYCLOIDEA/PCF (TCP) transcription factors are essential in regulating various development process. However, the role of TCPs in sunflowers has not yet been studied. This study, 34 *HaTCP* genes were identified and classified into three subfamilies based on the conservative domain and phylogenetic analysis. Most of the *HaTCPs* in the same subfamily displayed similar gene and motif structures. Promoter sequence analysis has demonstrated the presence of multiple stress and hormone-related cis-elements in the HaTCP family. Expression patterns of *HaTCPs* revealed several *HaTCP* genes expressed highest in buds and could respond to decapitation. Subcellular localization analysis showed that HaTCP1 was located in the nucleus. Paclobutrazol (PAC) and 1-naphthylphthalamic acid (NPA) administration significantly delayed the formation of axillary buds after decapitation, and this suppression was partially accomplished by enhancing the expression of *HaTCP1*. Furthermore, *HaTCP1* overexpressed in Arabidopsis caused a significant decrease in branch number, indicating that *HaTCP1* played a key role in negatively regulating sunflower branching.

**Conclusions:**

This study not only provided the systematic analysis for the HaTCP members, including classification, conserved domain and gene structure, expansion pattern of different tissues or after decapitation. But also studied the expression, subcellular localization and function of *HaTCP1*. These findings could lay a critical foundation for further exploring the functions of *HaTCPs*.

**Supplementary Information:**

The online version contains supplementary material available at 10.1186/s12870-023-04211-0.

## Background

Plant architecture is one of the essential characteristics of ornamental plants. Shoot branching, as the primary component of plant architecture, includes the initiation of axillary meristems and the growth of axillary buds [1]. Axillary meristems are initiated at the axils of leaves to form buds [2], which can grow into lateral branches or become dormant. The dormant axillary buds can also be reactivated and grow into lateral branches. Generally, bud fate is affected by many regulation factors, such as genes, hormones, and environmental factors [2]. Among them, numerous genes have been revealed to control plant branch development, with TCP being a critical family.

TCP genes are widely involved in plant growth and development, including seed germination, floral asymmetry, gametophyte development, leaf senescence, circadian rhythms, and defense responses [3]. TCP protein was named after the initial member TEOSINTE BRANCHED 1 (TB1), CYCLOIDEA (CYC) and rice PROLIFERATING CELL FACT ORS1(PCF1) and PCF2 [4]. TCP proteins can be divided into two classes based on the differences within the TCP domain. Class I is also called the PCF subfamily in angiosperms, and class II is further subdivided into the CIN clade and CYC/TB1 clade [4].

*BRC1*, the most famous member of the TCP family, is mainly involved in branch development [3]. *BRC1* is considered to be an integrator of several signalling networks, including hormonal, photochemical, and nutritional networks [5]. Auxin may unintentionally enhance bud *BRC1* expression [6]. High cytokinin (CK) levels downregulate *BRC1* expression, which activates axillary buds [7]. *BRC1* expression was upregulated by strigolactone (SL), and shoot branching in the *brc1* mutant was insensitive to SL [6, 8, 9], indicating *BRC1* acted downstream of SL. Active PHYB suppressed the expression of the *SbTB1* gene in sorghum, leading to high plant branching, and a low R/FR ratio favored *AtBRC1* upregulation. A slight decrease in the photosynthetic leaf area is associated with stimulation of *TB1* expression in sorghum seedlings and, consequently, a lower propensity of tiller buds to grow out [10]. All these findings indicate that *BRC1* expression is very sensitive to light intensity and quality. Applying sucrose to rose axillary buds reduced the expression level of *RhBRC1* [11]. Increasing the sucrose level in pea plants will significantly inhibit the expression of *BRC1* [5]. In addition, TIE1 can inhibit the transcriptional activity of *BRC1* from regulating shoot branching [12].

A crucial component of cut flowers is the sunflower. To ensure the quality of the top flower, it must remove buds repeatedly throughout cultivation, raising the cost of the final product. Therefore, producing new sunflowers with few or no branches is crucial. This study characterised the genes of the sunflower TCP family, and examined the evolutionary relationships, gene structures, conserved domains, cis-elements, gene expression patterns, subcellular localization, and function of *HaTCP1*. Our study lay the theoretical groundwork for further investigations into the roles of *HaTCP* genes in shoot branching.

## Results

### Identification and classification of TCP members in sunflower

Thirty-four sunflower TCP proteins were identified and named HaTCP1-HaTCP34. Protein sequence length, molecular weight (MW), and isoelectric point (IP) were all analyzed. The number of amino acids (aa) encoded by the TCP family genes was between 127 aa (HaTCP33) and 497 aa (HaTCP8), with an average of 322.9 aa. The MW of the TCP family proteins was between 14.21 (HaTCP33) and 53.42 KDa (HaTCP8). Subcellular localization predicted that TCP proteins were all localized in the nucleus (Table S1).

### Phylogenetic analysis of the TCP members

**Fig. 1:**
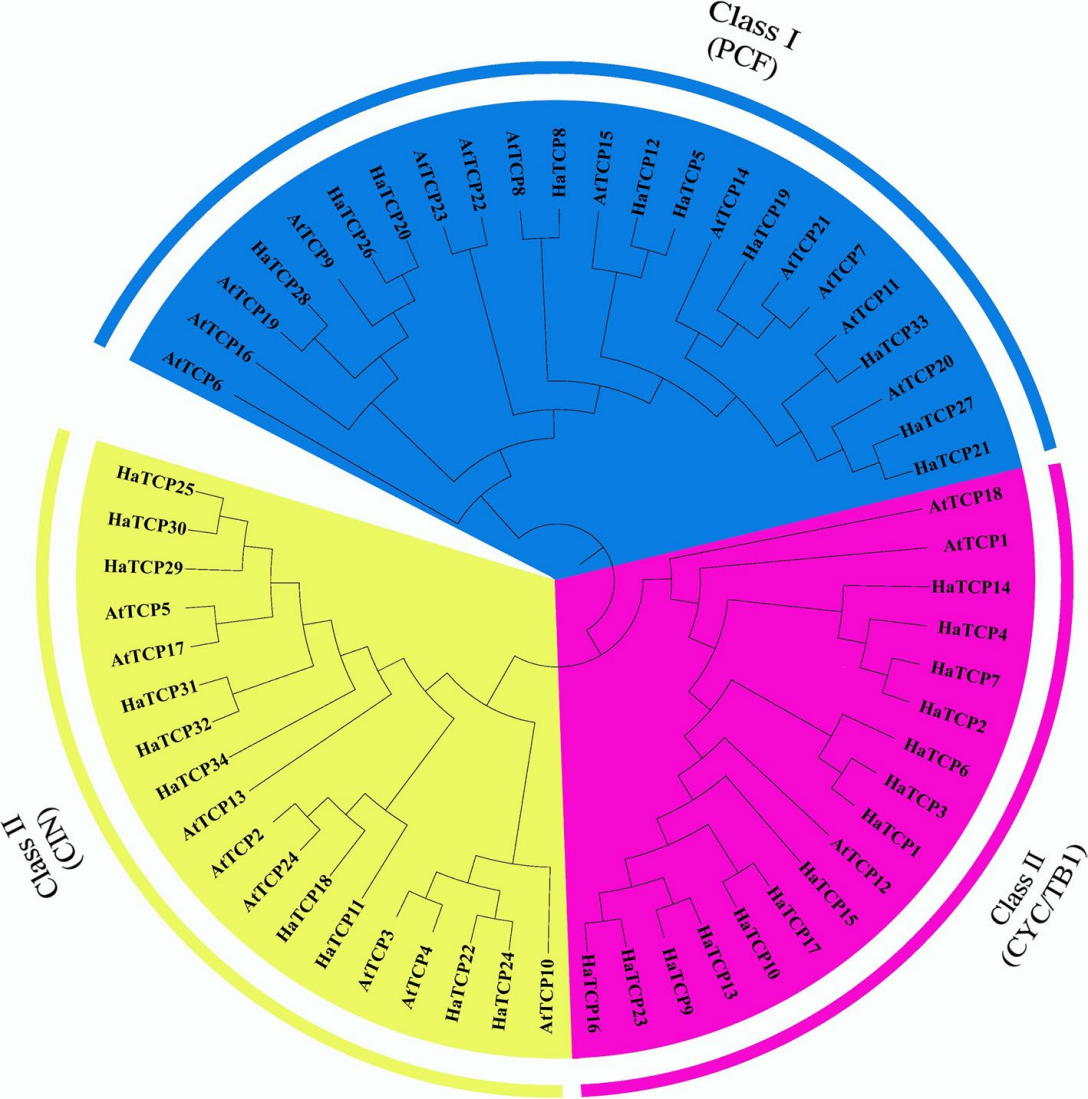
Evolutionary tree of TCPs

TCP proteins of the Arabidopsis and sunflower were all arranged into a phylogenetic tree to examine the evolutionary relationships between the various members of the sunflower TCP family. The TCP proteins from 24 Arabidopsis and 34 sunflowers revealed clear evolutionary grouping. As shown in Fig. 1, the TCP family can be divided into two subfamilies: class I and II. And class II TCP members were further divided into CIN and CYC/TB1 subfamilies [13]. The three subfamilies contained 10, 10, and 14 HaTCP members, respectively. CYC/TB1 accounts for 41.2% of the total HaTCP proteins. In this subfamily, AtTCP1, AtTCP12, and AtTCP18 all play a role in shoot branching, indicating that these *HaTCP* genes may also be involved in regulating shoot branching.

### Gene structure and conserved motifs of TCP in sunflower

**Fig. 2:**
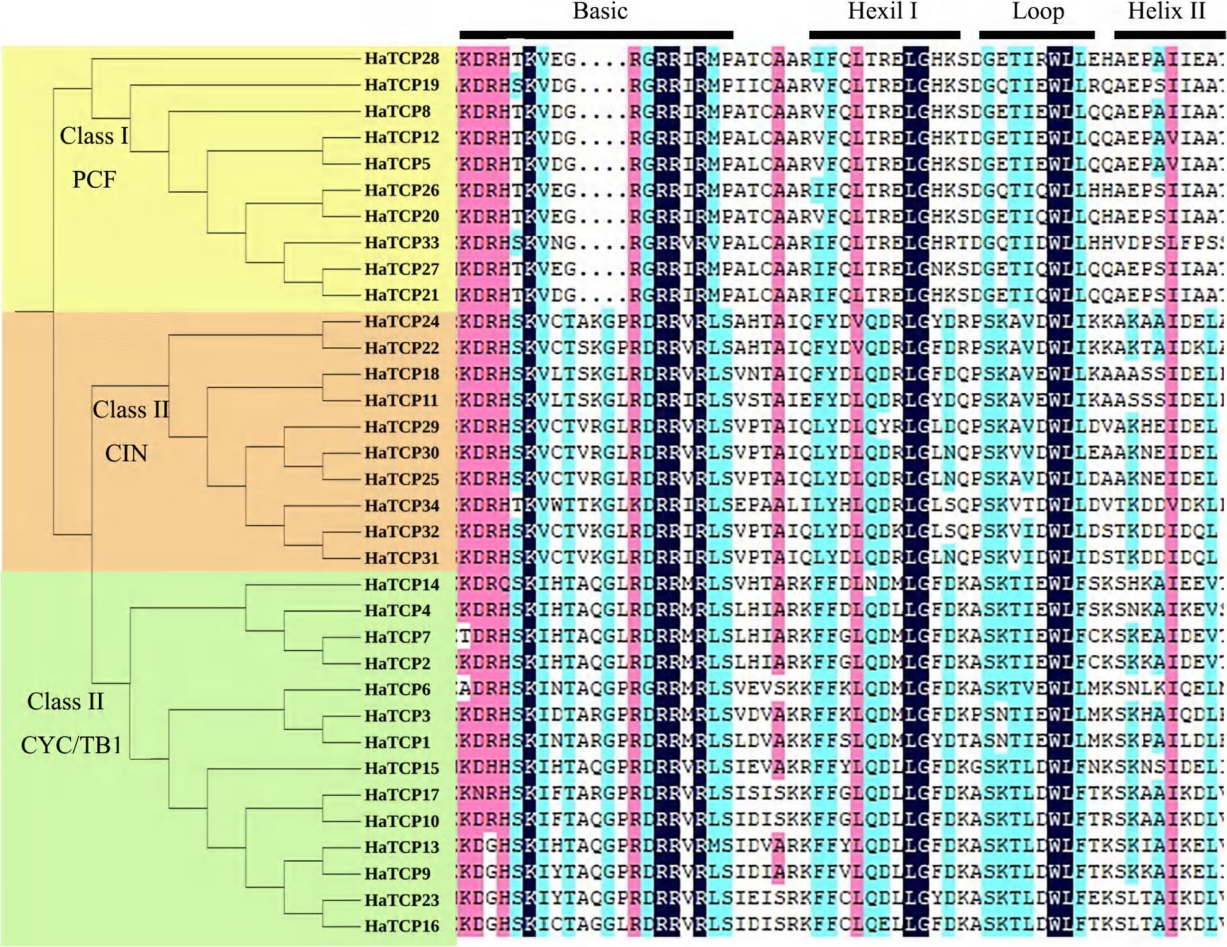
Sequence alignment of the conserved domains of TCP proteins in sunflower

We investigated the gene structure of *HaTCP* genes, and we found that all the *HaTCP* genes have no introns. To analyse the conservation and diversity of the TCP domain regions in HaTCP proteins, a multiple sequence alignment was performed using DNAMAN software based on the amino acid sequences of each TCP domain (Fig. 2). Alignment results showed that sunflower TCP proteins contained a typical TCP conservative domain: a bHLH domain, including a basic region, a loop region, and two helical regions (Helix I and II). Compared with class II, class I had four amino acid deletions in the basic region, which is similar to the structure of TCP proteins in other species, indicating the conservatism of TCP members in the evolution of different species.

**Fig. 3:**
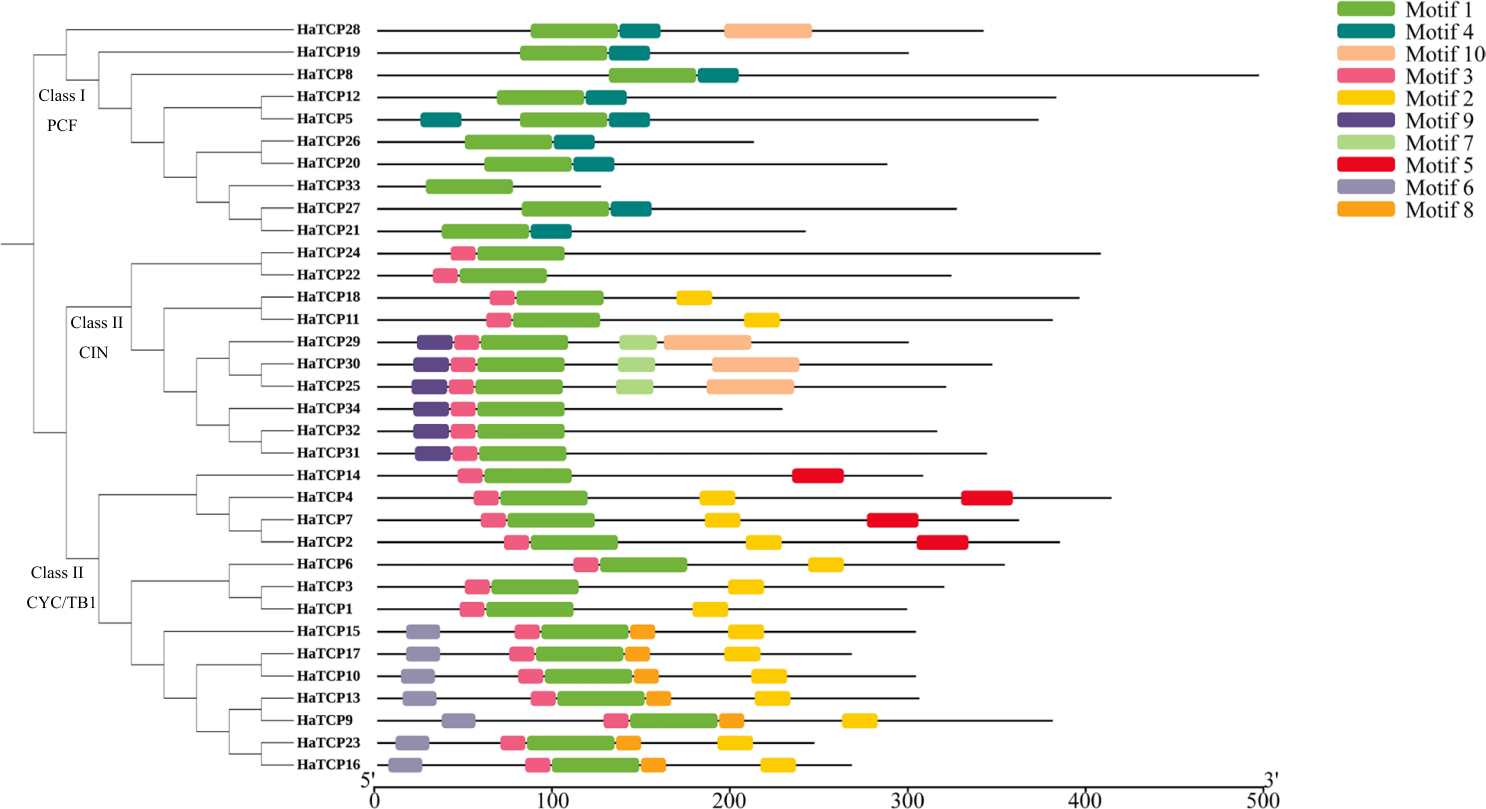
Analysis of the conserved motifs of HaTCP proteins

Next, we performed a conserved motif analysis of the HaTCP proteins using MEME software to observe their diversity of motif composition (Fig. 3). As seen in Fig. 3, a total of 10 conserved motifs were found. Motif 1 is a TCP conservative domain shared by all TCP proteins. Almost all the HaTCP proteins in the same branch of the evolutionary tree possess a similar motif composition. At the same time, significant differences can be seen in different branches, indicating that HaTCP members in the same branch could play similar roles. However, some motifs only exist in specific branches. For example, motif four and motif 3 only existed in class I or II, respectively, indicating that the genes possessing these motifs may perform particular functions.

### The Cis-elements in the promoters of TCP genes in sunflower

**Fig. 4:**
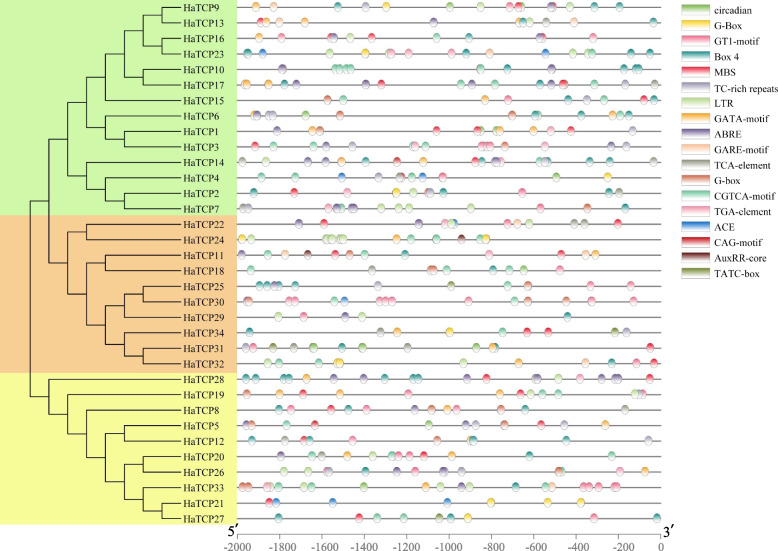
Cis-acting element analysis of promoters of *HaTCPs*

The 2000 bp promoter sequences of the *HaTCP* genes were extracted and submitted to the PlantCARE database to detect cis-acting elements. As depicted in Fig. 4, there were various hormone and stress response elements and many light response elements. Five hormone-related elements were auxin response elements (TGA-element, AuxRR-core), gibberellin (GA) response elements (GARE-motif, TATC-box), salicylic acid (SA) reaction element (TCA-element), jasmonic acid (JA) reaction element (CGTCA-motif), abscisic acid (ABA) reaction element (ABRE). Three elements involved in stress were defense and stress response element (TC-rich repeats), MYB binding sites (MBS) involved in drought induction, and cryogenic reaction element (LTR). The light-responsive element G-box were the most abundant elements in the promoter regions of 34 HaTCPs, with 33 promoters containing the element. GATA-motif was found in 21 promoters. Twenty-three *HaTCP* gene promoters had Box 4 and GT1-motif. CAG-motif, ACE, MREand AE-box were detected in 2, 5, 12, and 14 promoters of *HaTCP* genes, respectively. The above promoter analysis revealed that the expression of TCP family genes is widely involved inplant responses to environmental stress and hormones (Fig. 4).

### Expression analysis of different tissues of *HaTCP* genes

**Fig. 5:**
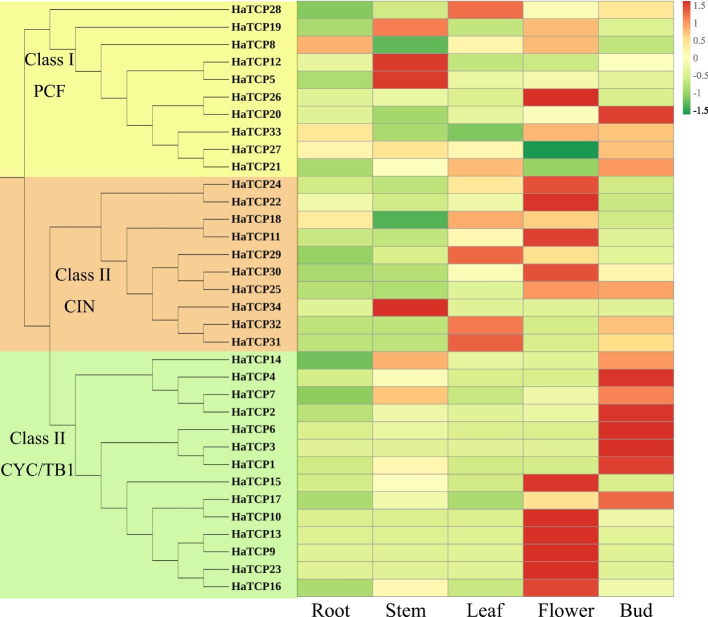
Tissue expression analysis of *HaTCP* genes

Based on transcriptome data from five tissues, including roots, stems, leaves, buds, and flowers, the expression profiles of *HaTCPs* were examined to investigate the potential roles of these genes in plant growth and development (Fig. 5). We detected the expression of 34 *HaTCP* genes, 11 *HaTCP* genes presented the highest expression level in flowers, indicating that they might play an essential role in sunflower flower development. *HaTCP12*, *HaTCP5*, and *HaTCP34* expressed the highest in the stem. *HaTCP20*, *HaTCP27*, *HaTCP21*, *HaTCP14*, *HaTCP4*, *HaTCP7*, *HaTCP2*, *HaTCP6*, *HaTCP3*, *HaTCP1* and *HaTCP17* in buds were higher than that in any other tissue, revealing the potential functions of these genes in shoot branching.

### Expression analysis of the *HaTCP* genes after decapitation

**Fig. 6:**
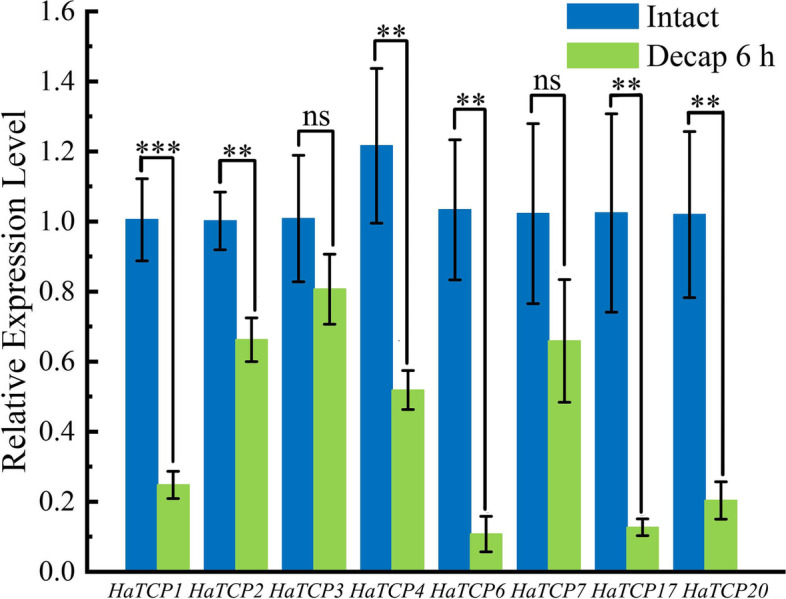
Relative expression analysis of *TCP* genes in sunflower. The significant differences are indicated by * (*p* < 0.05), ** (*p* < 0.01), *** (*p* < 0.001), ns (not significant)

We selected the genes with the highest expression level in axillary buds and detected the expression level of these genes. According to the Müller’s study, we chose 6 h after decapitation as the treatment time [14]. Our results demonstrated that, other genes, excluding *HaTCP3*, were all significantly downregulated, with *HaTCP1*, *HaTCP17*, and *HaTCP20* being downregulated to 0.24, 0.13, and 0.21 of the control, respectively (Fig. 6). These results suggest that these genes may play an important role in axillary bud germination after decapitation. Through similarity comparison, we found that *HaTCP1* and *AtBRC1* were closely related, indicating that *HaTCP1* is a homologous gene of *AtBRC1*. Therefore, we selected *HaTCP1* for further research.

### Subcellular localization analysis of HaTCP1

**Fig. 7:**
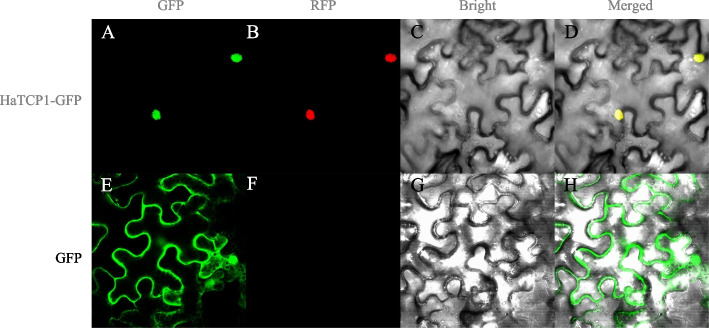
Subcellular localization analysis of HaTCP1. **A E** Green fluorescence images of HaTCP1-GFP protein and GFP (control). **B F** Red fluorescence image of marker for nucleus localization. **C G** Bright-field images of HaTCP1-GFP protein and control. **D H** The merged images of HaTCP1-GFP protein

The constructed HaTCP1-GFP vector was transformed into tobacco leaves, and the GFP signal was visualized under a laser confocal microscope. Figure 7 showed that both the HaTCP1-GFP green fluorescence signal and the red fluorescence signal of the nuclear marker were distributed in the nucleus, indicating that HaTCP1 was localized in the nucleus.

### Expression analysis of *HaTCP1* under different treatments

**Fig. 8:**
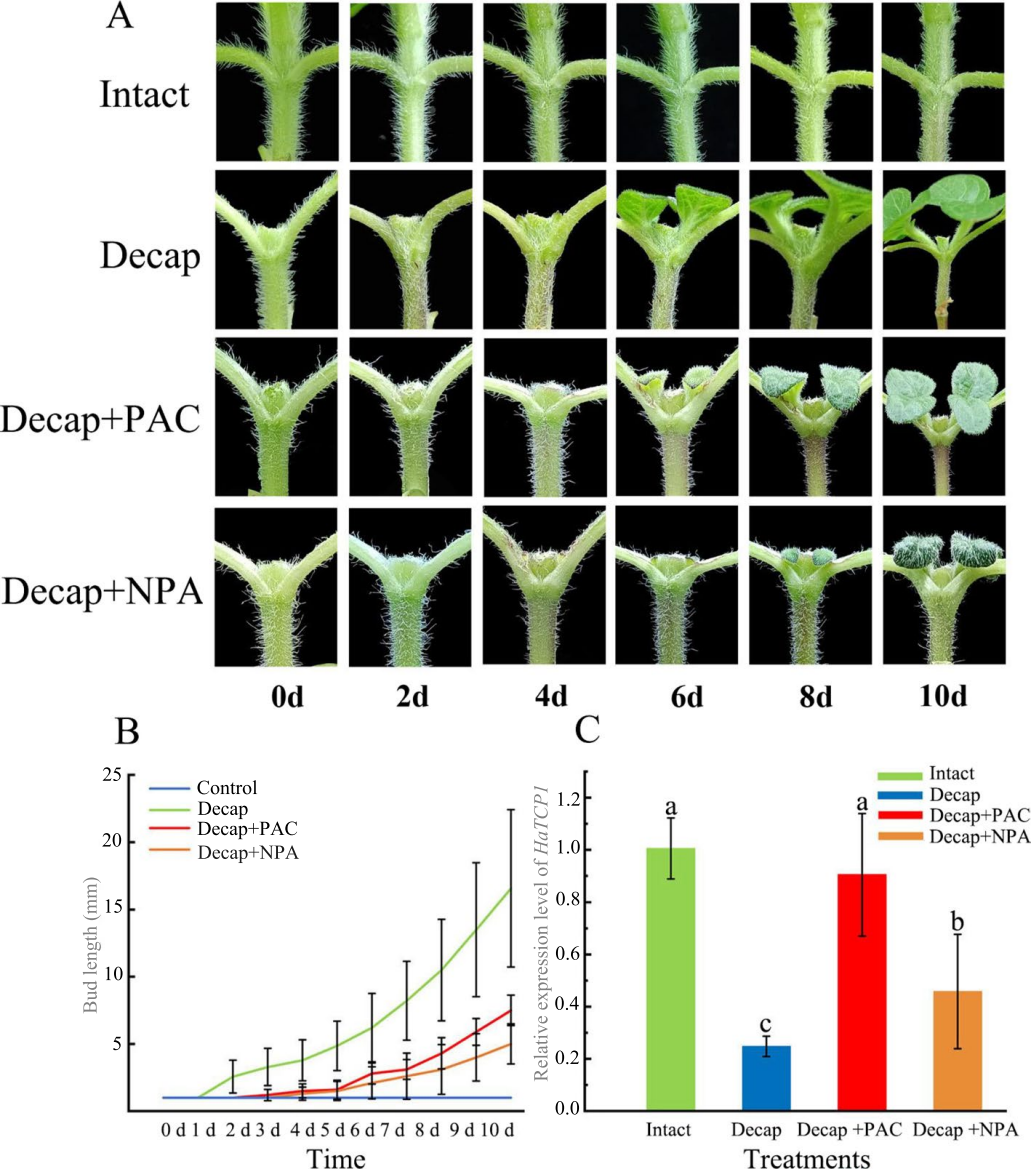
Axillary bud development and *HaTCP1* expression analysis induced by decapitation. **A** Axillary bud growth after 10 d of treatment. **B** Statistics of axillary bud length of sunflower after treatment for 10 d (*n* = 10). **C** The relative expression level of *HaTCP1* after 6 h of treatment. The data are mean ± SE

Solutions of gibberellin synthesis inhibitor (PAC) and auxin transport inhibitor (NPA) were given to the axillary buds of decapitated sunflower plants to study the effects of GA and auxin transport from the axillary bud to the stem on bud outgrowth (Fig. 8A). Then the relative expression of the *HaTCP1* under different treatments was examined using quantitative real-time PCR (qRT-PCR). The results showed that the axillary bud germinated after decapitation and reached 19 mm on the 10th day, while NPA and PAC significantly inhibited the axillary bud germination (Fig. 8B). This indicates that inhibiting auxin transport and GA synthesis can effectively retard axillary bud growth but can not wholly inhibit axillary bud germination. The treated axillary buds were sampled 6 h after the treatment, and the *HaTCP1* expression levels were detected by qRT-PCR. Application of PAC or NPA following decapitation both raised the level of *HaTCP1* expression compared to decapitation, showing that both treatments' effects on the development of axillary buds were caused, at least in part, by controlling the expression of this gene (Fig. 8C).

### *HaTCP1* inhibits shoot branching

**Fig. 9:**
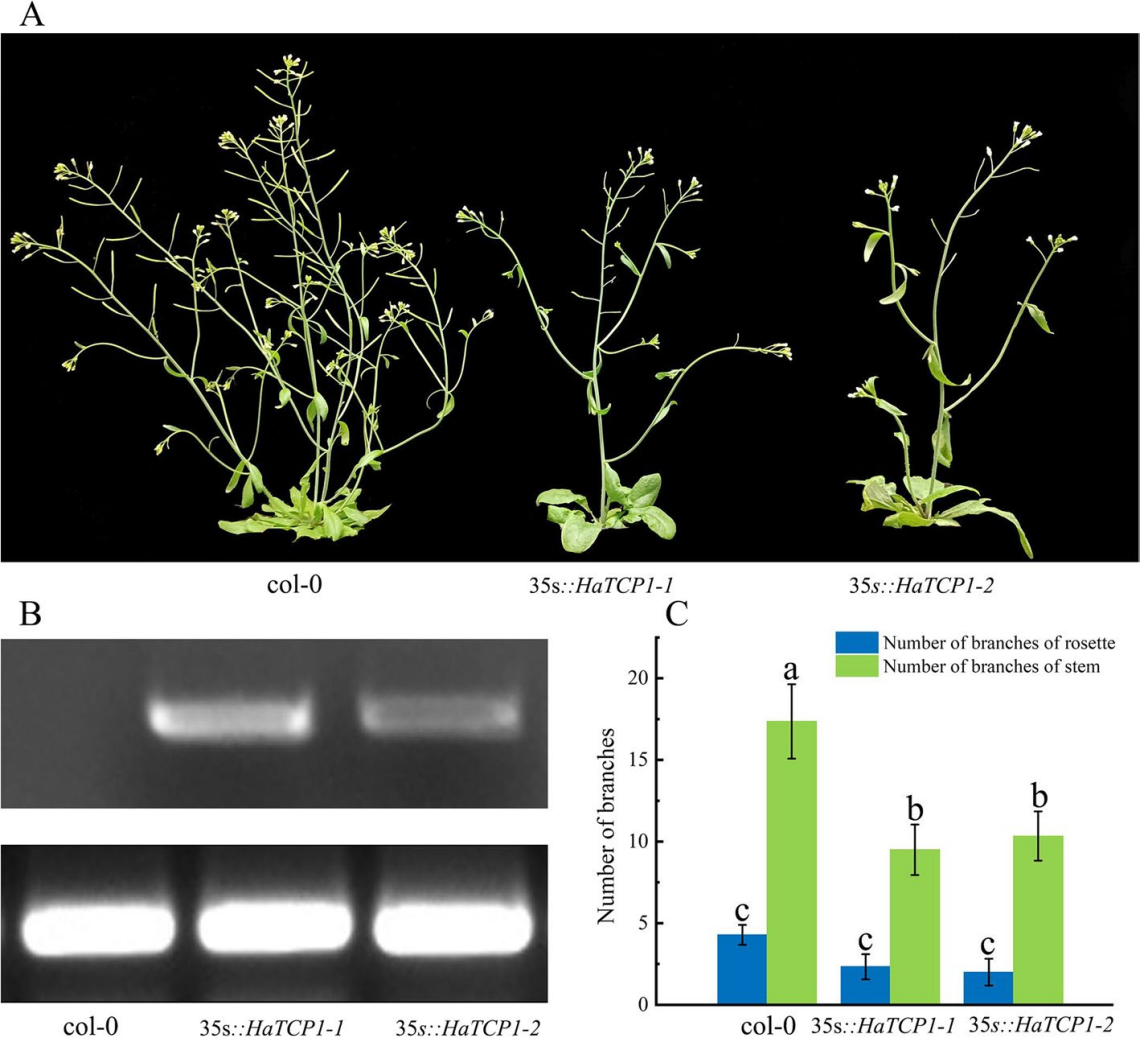
Analysis of the phenotype and gene expression in transgenic *HaTCP1*-overexpressing Arabidopsis plants. **A** Transgenic and Col-0 Arabidopsis plants. **B** In-gel electrophoresis detection of *HaTCP1* expression from the leaves of Arabidopsis Col-0 plants and *HaTCP1*-overexpressing transgenic plants. **C** Primary rosette or stem branches per plant were counted (*n* = 15)

To verify the *HaTCP1* function, ten Arabidopsis-overexpression lines were generated. Two separate transgenic lines were selected for observation and statistical analysis further to analyze the levels of gene expression and shoot branching. Semi-quantitative PCR analysis confirmed high *HaTCP1* gene expression in overexpression lines compared to Columbia-0 (Col-0) plants (Fig. 9B). We noticed that *HaTCP1* overexpression resulted in fewer stem and rosette branches in 38-day-old seedlings compared to the Col-0 (Fig. 9A, C). In contrast to the Col-0, which had 4.3 rosette branches, the two transgenic lines had an average of 2 and 2.3 rosette branches each. Compared to the Col-0, which had 17.4 stem branches, transgenic overexpression lines had 9.5 and 10.3 stem branches, respectively. These results indicate that *HaTCP1* negatively regulates shoot branching in sunflowers.

## Discussion

Plant-specific transcription factors TCPs play various roles in plant growth and development aspects. Although the TCP transcription factors have been identified in a wide range of plant species, such as Arabidopsis [15], rice [15], tomato [16], tobacco [17], and strawberry [18], little is known in sunflowers. In this study, we identified 34 putative *HaTCP* genes. Like other species, HaTCPs were divided into three subfamilies: PCF, CYC/TB1, and CIN [15].

Different types of TCP transcription factors have other functions. Studies showed that class II TCP members controlled stem branching [5, 19]. The CIN subclade TCP genes interfere with several cellular pathways controlling leaf development [20]. Class I (PCF) TCP factors primarily induce cell division and promote plant growth [4]. Therefore, these HaTCP members belonging to different subfamilies may have corresponding functions.

We analysed the distribution of motifs and found that HaTCP members from the same group or subgroup shared a similar motif composition. In class I and class II subgroups alone, for instance, motif 4 and motif 3 were found (Fig. 3). The hydrophobic amphiphilic helix (the first helix) may control protein–protein interaction through the *HaTCP* genes, allowing for the formation of homologous and heterologous dimers [21]. However, there are structural differences between classes I and II, such as the four amino acid deletions in the basic region of class I members. The consistency of the motif compositions and gene structures of *HaTCP* genes further supported the close evolutionary relationships.

Most of the TCPs have cis-regulatory elements related to light responses (AE-box, BOX4, GBOX, GT1-motif and GATA-motif). Furthermore, the different TCP members possessed other hormone-related characteristics, such as ABRE (ABA), AuxRR-core/TGA-element (auxin), CGTCA-motif (MeJA), GARE-motif/TATC-box (gibberellin), and TCA-element (SA). These results indicated that the *HaTCP* genes might influence response to plant hormones.

The TCP genes are involved in plant growth and development and could respond to multiple abiotic stresses. The analysis of promoter regions showed that some TCPs contained TC-rich repeats, LTR cis-regulatory elements, and MBS cis-regulatory elements, suggesting that the *HaTCP* genes might play a significant role in stress responses.

Gene expression patterns provide important information related to gene functions [22]. Among the genes expressed highest in leaves, *HaTCP29*, *HaTCP31*, and *HaTCP32* are closely related to *AtTCP5*. However, *AtTCP5* was proven to be involved in controlling leaf margin development [23], indicating that *HaTCP29*, *HaTCP31*, and *HaTCP32* may play an essential role in regulating the development of sunflower leaves. *HaTCP12* and *HaTCP5* showed the highest expression in stems, and the two genes are closely related to *AtTCP5*. Previous studies have shown that *TCP14* and *TCP15* affect internode length in Arabidopsis [24], indicating that *HaTCP12* and *HaTCP5* might be involved in regulating the development of sunflower stems.

Application of NPA inhibited the initiation of AMs in the maize inflorescence [25]. The mutation of *PIN-FORMED1* (*PIN1*) resulted in a pin-like stem architecture [26]. AM formation is strongly compromised in the polar auxin transport mutant *barren inflorescence2* (*bif2*) [27, 28]. These studies indicate that transport is required for axillary meristem formation.

Some studies also showed that NPA did not affect the initialbud outgrowth after decapitation but only affected the growth of axillary buds after germination [29]. Our study found that applying NPA after sunflower decapitation significantly delayed the germination of axillary buds but did not wholly prevent the germination of axillary buds.

The role of gibberellin in branching has remained obscure. GA is often viewed as a branching inhibitor because GA-biosynthesis and -perception mutants exhibit more branches [30]. At the same time, studies in Jatropha suggested that GA application promotes bud outgrowth [31]. According to our research findings, endogenous gibberellin was essential for the proper germination of axillary buds since its inhibition significantly slowed the germination of axillary buds. The detection of *HaTCP1* expression level revealed that the application of NPA and PAC significantly promoted the up-regulation of *HaTCP1* expression level, indicating that the inhibition of auxin transport and gibberellin synthesis was at least partially mediated by regulating the expression of *HaTCP1*, thereby inhibiting the growth of axillary buds.

Many TCPs, such as TEOSINTE BRANCHED1 (TB1) from maize, Arabidopsis *BRC1* and *BRC2*, and rice PROLIFERATING CELL FACTOR, were all involved in plant branching [3]. The ectopic overexpression of these genes significantly reduced lateral branching [32]. Similarly, HaTCP1 is located in the nucleus and features typical transcription factor characteristics, and its overexpression in Arabidopsis decreased the number of rosette branches and stem branches. The expression analysis of *HaTCP1* under different treatments and the phenotype of Arabidopsis plants overexpressed *HaTCP1* showed that *HaTCP1* could negatively regulate sunflower branching.

## Conclusion

In summary, 34 *TCP* genes were identified in sunflowers and were divided into three subfamilies. A comprehensive analysis of phylogenetic relationships, conserved motifs, and expression profiles was also performed. We observed similar exon–intron structures and protein motif distribution patterns for *HaTCP* genes. Expression analysis of sunflower TCP family genes in different tissues revealed that *HaTCP* genes showed strong tissue specificity. Decapitation causes a significant decrease in the expression level of *HaTCP1*, *HaTCP17*, and *HaTCP20*. In addition, NBA and PAC significantly inhibited the germination speed of axillary buds in sunflowers, which is related to the change in the *HaTCP1* expression level. The overexpression of *HaTCP1* significantly reduced the number of Arabidopsis stem branches and rosette branches. Our study lays a solid foundation for future sunflower TCP function exploration and provides a reference for *TCP* gene studies.

## Methods

### Plant materials

Seeds of *Helianthus annuus* cv ‘huoli’, *Arabidopsis thaliana* Columbia-0 (Col-0) and transgenic plants, *Nicotiana benthamiana*, were sown in pots (5 × 5 cm) and placed in an artificial climate chamber. The light intensity is 5500 LX, the light cycle is 16/8 h (light/dark), and the temperature is 20 ± 2 ℃. All plant materials were grown in feld of plant growth chamber, College of Horticulture, Anhui Agricultural University, Hefei, Anhui.

### Identification of *TCP* genes in sunflower

The whole genome sequence of sunflower was downloaded from the ensemble database (ftp://ftp.ensemblgenomes.org/pub/plants/release-44/fasta/helianthus_annuus/). The sunflower TCP family genes were identified through the simple HMM search tool of Tbtools software. After deleting repetitive sequences and genes not containing the TCP domain, the TCP family members were finally determined. The physical and chemical properties of sunflower TCP proteins, including molecular weight (MW), protein sequence length and isoelectric point (IP), were assessed with the online software ExPASY (http://web.expasy.org/protparam/). To predict the subcellular localization of the identified HaTCP proteins, WoLF PSORT (https://wolfpsort.hgc.jp/) was used.

### Phylogenetic analysis of the TCP proteins

The TCP protein sequences of sunflower and Arabidopsis were compared by MEGA 11 software. The neighbour-joining method constructed the phylogenetic tree with a boot-strap value based on 1000 replicates. Arabidopsis TCP protein sequences were obtained from the UniProt protein database (https://www.uniprot.org/).

### Analysis of motifs and gene structure

Te conserved motifs for each HaTCP member were analyzed using MEME Suite version 5.0.5 (http://meme.nbcr.net/meme/). The gene structures of the *HaTCPs* were determined by the online Gene Structure Display Server (GSDS) 2.0 software (http://gsds.cbi.pku.edu.cn/).

### Analysis of Cis-elements in promoters

The upstream 2000 bp sequence of *HaTCP* genes in the sunflower genome was extracted. The plant care online website (http://bioinformatics.psb. ugent.be/webtools/plantcare/html/) was used to predict plant cis-acting elements, and the predicted results were drawn out using the Basic Biosequence View tool of Tbtools.

### Expression analysis of *HaTCP* genes in different tissues or under different treatments

The tissue-specific expression analysis was performed according to the method reported by Dong et al. [33]. For the decapitation, NPA and PAC experiment, thirty-day-old seedlings of *H. annuus* cv ‘huoli’ were divided into four groups. One group was used as intact, the second group was decapitated, and the third and fourth groups were treated with 10 ul NPA (1 M) or PAC (100 mM) solution after decapitation. Take photos of the treated axillary buds every other day and measure the length of the axillary buds. Take samples of axillary bud at 0 or 6 h after treatments. The expression level of *HaTCP1* or other genes was detected by qRT-PCR, and *eF1A* [34]. was employed as the internal control (Table S2).

### Subcellular localization

The coding regions of *HaTCP1* were amplified with primers TCP1-F/TCP1-R. Vector construction, tobacco injection and fluorescence observation were carried out according to the method reported by Dong et al. [33].

### Overexpression of *HaTCP1* and phenotype analysis in arabidopsis

The 35S::HaTCP1 was transformed to Arabidopsis according to the method reported by Dong et al. [33]. The expression level of *HaTCP1* was detected by qRT-PCR. The number of branches with a bud length ≥ 10 mm was scored, and the height of the main stems were measured for phenotype analysis.

### Statistical analysis

All the data in this study was presented as mean value ± SD. Tukey’s tests were performed for statistical analyses.

### Plants were sourced as follows

1. *Arabidopsis thaliana* (Col-0) was obtained from NASC (NASC ID: N1092) and grown continuously in the Laboratory of Associate professor Lili Dong. It was verifed by Associate professor Lili Dong from her seed stock registrar and confrmed by visual examination of plants that are grown for seed stocks.
2. *Helianthus annuus* cv‘huoli’ and *Nicotiana benthamiana* were obtained and verifed by Associate professor Lili Dong after cultivation at the laboratory in Anhui Agricultural University.

## Supplementary Information

**Additional file 1.**

**Additional file 2: Table S1.** Physicochemical properties of TCP members in Sunflower.

**Additional file 3: Table S2.** Primer sequences.

## Data Availability

All data supporting the findings of this study are available within the paper and within its supplementary materials published online. “The datasets generated and/or analysed during the current study are available in the Gene Expression Omnibus (GEO) repository, GSE221055 https://www.ncbi.nlm.nih.gov/geo/query/acc.cgi?acc=GSE221055.
