# Supplementary material for: Genome-wide analysis of TCP transcription factor family in sunflower and identification of HaTCP1 involved in the regulation of shoot branching
Source: BMC Plant Biol. 2023 Apr 27;23:222. doi: 10.1186/s12870-023-04211-0 (PMC10134548; doi:10.1186/s12870-023-04211-0)
Supplement: Supplementary file 1 — Additional file 1. [file 12870_2023_4211_MOESM1_ESM.docx]

Supplementary Figure 9B.


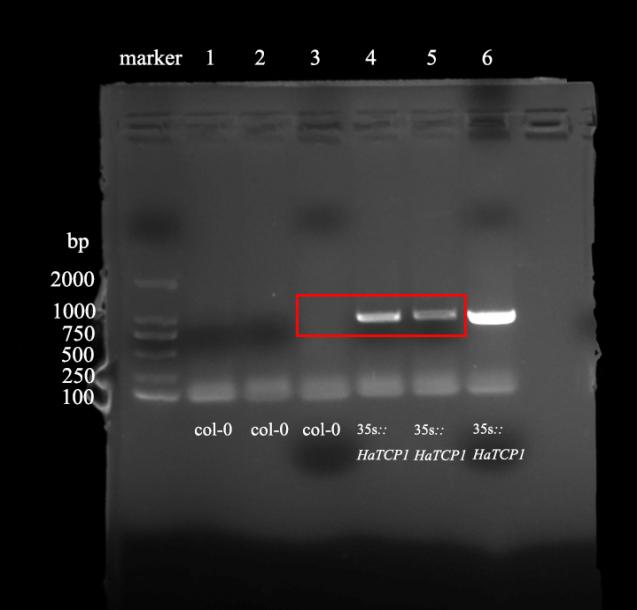


In-gel electrophoresis detection of *HaTCP1* expression from the leaves of Arabidopsis Col-0 plants and HaTCP1-overexpressing transgenic plants.


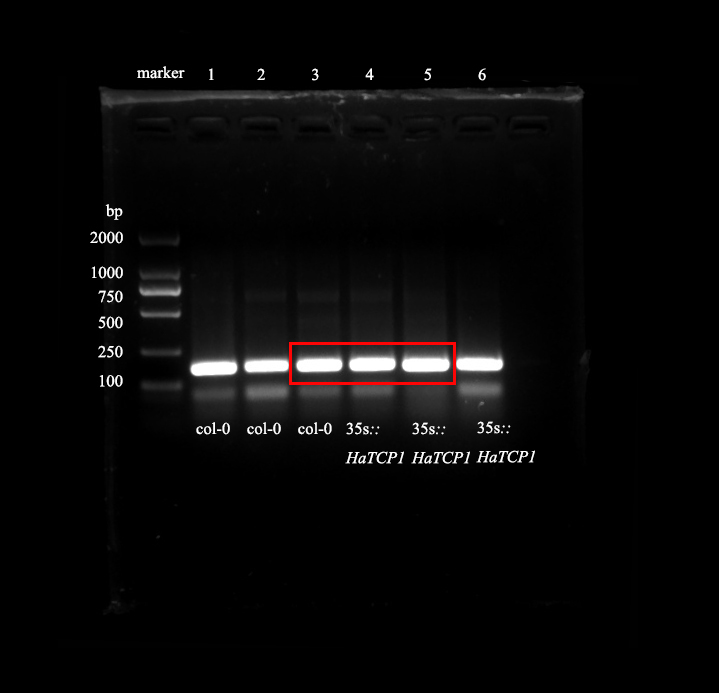
In-gel electrophoresis detection of *Tubulin* expression from the leaves of Arabidopsis Col-0 plants and HaTCP1-overexpressing transgenic plants.
