# Supplementary material for: Genome-wide analysis of TCP transcription factor family in sunflower and identification of HaTCP1 involved in the regulation of shoot branching
Source: BMC Plant Biol. 2023 Apr 27;23:222. doi: 10.1186/s12870-023-04211-0 (PMC10134548; doi:10.1186/s12870-023-04211-0)
Supplement: Supplementary file 2 — Additional file 2: Table S1. Physicochemical properties of TCP members in Sunflower. [file 12870_2023_4211_MOESM2_ESM.docx]

| Gene name | Gene ID | Subcellular localization | Protein properties | | | |  |
| --- | --- | --- | --- | --- | --- | --- | --- |
|  |  |  | Amino acid | Molecular weight | Isoelectric point | Instability coefficient | |
| HaTCP1 | HannXRQ_Chr16g0501831 | nucleus | 299 | 34145.22 | 8.94 | 46.62 | |
| HaTCP2 | HannXRQ_Chr05g0149701 | nucleus | 385 | 43538.92 | 6.96 | 55.41 | |
| HaTCP3 | HannXRQ_Chr09g0269811 | nucleus | 320 | 36531.22 | 9.05 | 47.89 | |
| HaTCP4 | HannXRQ_Chr12g0365351 | nucleus | 414 | 46671.33 | 6.04 | 54.63 | |
| HaTCP5 | HannXRQ_Chr05g0158111 | nucleus | 373 | 39993.75 | 7.99 | 43.97 | |
| HaTCP6 | HannXRQ_Chr05g0153871 | nucleus | 354 | 39850.27 | 9.06 | 52.18 | |
| HaTCP7 | HannXRQ_Chr16g0528881 | nucleus | 362 | 41317.82 | 5.70 | 57.46 | |
| HaTCP8 | HannXRQ_Chr10g0294511 | nucleus | 497 | 53420.60 | 6.62 | 55.47 | |
| HaTCP9 | HannXRQ_Chr04g0112191 | nucleus | 381 | 43569.87 | 7.66 | 46.16 | |
| HaTCP10 | HannXRQ_Chr10g0283781 | nucleus | 304 | 34605.02 | 9.70 | 42.11 | |
| HaTCP11 | HannXRQ_Chr15g0482041 | nucleus | 381 | 41602.44 | 9.01 | 45.64 | |
| HaTCP12 | HannXRQ_Chr16g0502851 | nucleus | 383 | 40619.76 | 6.78 | 58.80 | |
| HaTCP13 | HannXRQ_Chr15g0489921 | nucleus | 306 | 35261.74 | 9.21 | 50.48 | |
| HaTCP14 | HannXRQ_Chr12g0365361 | nucleus | 308 | 34560.59 | 7.75 | 45.14 | |
| HaTCP15 | HannXRQ_Chr04g0112141 | nucleus | 304 | 34665.42 | 5.90 | 54.68 | |
| HaTCP16 | HannXRQ_Chr08g0209331 | nucleus | 268 | 30401.68 | 7.65 | 46.95 | |
| HaTCP17 | HannXRQ_Chr15g0472911 | nucleus | 268 | 30428.57 | 9.81 | 44.27 | |
| HaTCP18 | HannXRQ_Chr16g0525741 | nucleus | 396 | 43913.24 | 9.17 | 61.39 | |
| HaTCP19 | HannXRQ_Chr17g0541411 | nucleus | 300 | 31852.78 | 9.66 | 57.94 | |
| HaTCP20 | HannXRQ_Chr04g0122181 | nucleus | 288 | 31255.81 | 8.74 | 56.74 | |
| HaTCP21 | HannXRQ_Chr07g0198131 | nucleus | 242 | 25798.72 | 6.58 | 56.24 | |
| HaTCP22 | HannXRQ_Chr12g0373501 | nucleus | 324 | 35720.45 | 8.76 | 52.44 | |
| HaTCP23 | HannXRQ_Chr15g0489241 | nucleus | 247 | 28250.75 | 8.21 | 47.52 | |
| HaTCP24 | HannXRQ_Chr10g0304701 | nucleus | 408 | 44686.55 | 6.03 | 51.29 | |
| HaTCP25 | HannXRQ_Chr17g0546041 | nucleus | 321 | 36133.90 | 6.15 | 51.76 | |
| HaTCP26 | HannXRQ_Chr06g0169341 | nucleus | 213 | 23794.85 | 9.92 | 56.36 | |
| HaTCP27 | HannXRQ_Chr09g0256441 | nucleus | 327 | 35021.62 | 7.92 | 48.01 | |
| HaTCP28 | HannXRQ_Chr15g0486311 | nucleus | 342 | 36150.20 | 5.42 | 54.16 | |
| HaTCP29 | HannXRQ_Chr16g0522201 | nucleus | 300 | 33481.09 | 6.45 | 61.94 | |
| HaTCP30 | HannXRQ_Chr15g0472431 | nucleus | 347 | 38683.76 | 7.16 | 46.78 | |
| HaTCP31 | HannXRQ_Chr09g0270421 | nucleus | 344 | 38563.05 | 8.39 | 48.89 | |
| HaTCP32 | HannXRQ_Chr16g0502471 | nucleus | 316 | 35572.06 | 7.80 | 42.21 | |
| HaTCP33 | HannXRQ_Chr13g0396671 | nucleus | 127 | 14212.00 | 6.64 | 61.84 | |
| HaTCP34 | HannXRQ_Chr08g0230441 | nucleus | 229 | 26251.60 | 6.86 | 44.87 | |

Table S1 Physicochemical properties of TCP members in Sunflower
