# Supplementary material for: Genome-wide analysis of TCP transcription factor family in sunflower and identification of HaTCP1 involved in the regulation of shoot branching
Source: BMC Plant Biol. 2023 Apr 27;23:222. doi: 10.1186/s12870-023-04211-0 (PMC10134548; doi:10.1186/s12870-023-04211-0)
Supplement: Supplementary file 3 — Additional file 3: Table S2. Primer sequences. [file 12870_2023_4211_MOESM3_ESM.docx]

Table S2 Primer sequences

| Primer name | Primer sequences |
| --- | --- |
| HaTCP1-F | ATGCATCCTTCCTACATTAACAG |
| HaTCP1-R | CTAATTGTTGATGTCTTCCCATG |
| HaTCP1-1300-F | gagaacacgggggactctagaATGCATCCTTCCTACATTAACAG |
| HaTCP1-1300-R | gcccttgctcaccatggatccATTGTTGATGTCTTCCCATGGTT |
| HaTCP1-qRT-F | ACCCGCTATTCTAGATCTCC |
| HaTCP1-qRT-R | AACACTTTTCCTCACTCCTC |
| HaTCP2-qRT-F | GGCAGTTTCAGACTCCTC |
| HaTCP2-qRT-R | CTTTCTTGCACGTTTAGTT |
| HaTCP3-qRT-F | ATGTCAAGCGATTGGAGC |
| HaTCP3-qRT-R | CAAATCACTGAACGGAGG |
| HaTCP4-qRT-F | ACAAACCGCTAAATGTCA |
| HaTCP4-qRT-R | ATCTTGCTGTGCCTATCT |
| HaTCP6-qRT-F | ATGTATCCCTCTTTCAC |
| HaTCP6-qRT-R | ACAGTAATCTTCGCAAT |
| HaTCP7-qRT-F | GCAGTTCCCGTCTCCTT |
| HaTCP7-qRT-R | GTCCGTCTTTCCAGCAC |
| HaTCP17-qRT-F | TACTTTAACCACCACCC |
| HaTCP17-qRT-R | TTTGAAGCAGCAACTCT |
| HaTCP20-qRT-F | ACACGAAAGTTGAGGGA |
| HaTCP20-qRT-R | AAGGTGCCGTTTACTGA |
| eF1A-F | AGCCCAAGAGACCCTCAGACAAG |
| eF1A-R | CCCTGATGGTCCGAAGGTAACAAC |
